# Characterisation of a Marine Bacterium *Vibrio Brasiliensis* T33 Producing *N*-acyl Homoserine Lactone Quorum Sensing Molecules

**DOI:** 10.3390/s140712104

**Published:** 2014-07-08

**Authors:** Wen-Si Tan, Nina Yusrina Muhamad Yunos, Pui-Wan Tan, Nur Izzati Mohamad, Tan-Guan-Sheng Adrian, Wai-Fong Yin, Kok-Gan Chan

**Affiliations:** Division of Genetics and Molecular Biology, Institute of Biological Sciences, Faculty of Science, University of Malaya, Kuala Lumpur 50603, Malaysia; E-Mails: tmarilyn36@gmail.com (W.-S.T); ninayusrina@hotmail.com (N.Y.M.Y.); acelinetan38@yahoo.com (P.-W.T.); zetty_mohamad@yahoo.com (N.I.M.); adrian_tan_1991@yahoo.com (T.-G.-S.A); yinwaifong@yahoo.com (W.-F.Y.)

**Keywords:** 16S rDNA, biosensor, *Chromobacterium violaceum* CV026, marine water-borne, *N*-acyl homoserine lactone (AHL), quorum sensing (QS), *N*-hexanoyl-l-homoserine lactone (C6-HSL), *N*-(3-oxo-decanoyl)-l-homoserine lactone (3-oxo-C10 HSL), *Vibrio brasiliensis*

## Abstract

*N*-acylhomoserine lactones (AHL) plays roles as signal molecules in quorum sensing (QS) in most Gram-negative bacteria. QS regulates various physiological activities in relation with population density and concentration of signal molecules. With the aim of isolating marine water-borne bacteria that possess QS properties, we report here the preliminary screening of marine bacteria for AHL production using *Chromobacterium violaceum* CV026 as the AHL biosensor. Strain T33 was isolated based on preliminary AHL screening and further identified by using 16S rDNA sequence analysis as a member of the genus *Vibrio* closely related to *Vibrio brasiliensis*. The isolated *Vibrio* sp. strain T33 was confirmed to produce *N*-hexanoyl-l-homoserine lactone (C6-HSL) and *N*-(3-oxodecanoyl)-l-homoserine lactone (3-oxo-C10 HSL) through high resolution tandem mass spectrometry analysis. We demonstrated that this isolate formed biofilms which could be inhibited by catechin. To the best of our knowledge, this is the first report that documents the production of these AHLs by *Vibrio brasiliensis* strain T33.

## 1. Introduction

The discovery of luminescence in the marine bacterium *Vibrio fischeri* in the late 1970s has paved the way for research into the mechanisms of regulation of bacterial physiological activities by cell-cell communication [1]. It was later shown that the luminescence was initiated not by inhibitor removal, but rather by accumulation of activator molecules, simply known as “autoinducers”. The bacteria are able to sense the cell density population by tracking the concentration of signal molecules. This phenomenon is named “quorum sensing (QS)” by Fuqua and co-workers [2,3]. In QS, the concentration of signal molecules plays a vital role that reflects the bacterial population density, when at quorate, specific target genes are activated [4] and a collective behavioral adaptation will occur [5].

There are three documented archetypal QS systems: the *N*-acyl homoserine lactone (AHL)-based signaling system of Gram-negative bacteria, an oligopeptide-based system in Gram-positive bacteria and a furanone-based system that is shared between both [6,7]. The AHL-based QS system has garnered significant interest because of its frequent role in microbial virulence mechanisms. QS is considered to be important as disruption of bacterial communication could be a strategy for developing potential antimicrobial therapeutic targets [3,8]. The cell density sensing apparatus utilized by the AHL-based QS system in most Gram-negative bacteria is the LuxI-LuxR system [9] where “LuxR” is a transcriptional activator protein [10] that functions by binding to a cognate signal molecule produced by “LuxI” autoinducer synthase which will result in specific target gene regulation [11]. The QS system is much more appreciated in the microbial world due to its capability in regulating different physiological activities such as competence, motility, development, antibiotic synthesis, virulence factor induction and cell differentiation [12,13].

To date, different marine bacteria have been studied and their QS properties portrayed and majority of these marine bacteria belongs to the genus *Vibrio* [14]. Several *Vibrio* species can be pathogenic, causes food-borne infections usually related with the consumption of undercooked seafood [15]. Most disease-causing strains are associated with gastroenteritis and it also can infect open wounds and cause septicemia [15]. For example, in the so-called Kanagawa phenomenon, hemolytic *V. parahaemolyticus* strains was isolated from human hosts on blood agar. *Vibrio* is considered as opportunistic pathogen where *V. vulnificus* typically infects the bloodstream and could cause life-threatening illnesses in persons with liver disease or immune-compromising conditions [16]. The pathogenic effect is believed to influenced by regulation of virulence determinants throughout the infection process and QS provide a means for these pathogenic bacteria to make a concerted attack and produce ample virulence factors to overwhelm the host defenses [13]. Research on QS pathogens could be significant in controlling disease besides helping understand their mechanism of pathogenicity. Therefore, this raised significant interest in our group to extend our research on isolating marine bacteria that possess QS properties. In this study, we investigated the presence of QS bacteria in a Malaysian tropical marine water sample. Here, we report the isolation of a *Vibrio* sp. QS bacterium, and its unique AHL production which has not been reported before.

## 2. Experimental Section

### 2.1. Water Sample Collection and Bacterial Strain Isolation

The sampling site chosen for this study was Morib Beach, Malaysia, with GPS coordinates of N 02°45.023′ E 101°26.623′. The water sample was collected along the beach coastal area in 2013. The sample was collected at a depth of 20 cm below the water surface and kept in a sterilized plastic bottle that was transferred at 4°C to the laboratory for analysis [17]. A serial dilution of water sample was carried out with sterile saline [18] and it was then spread onto Luria Bertani (LB) agar medium (in grams per 1 L: tryptone, 10; yeast extract, 5; NaCl, 30; Bacto agar, 15). Next, the plates were incubated overnight (24 h) at 28 °C. The observable colonies formed with different morphologies were isolated and pure colonies obtained by repeated streaking on LB medium.

### 2.2. Bacteria Strains, Culture Conditions and Biosensors Assay

The strain T33 was selected for further work after pure colonies were obtained and it was routinely cultured on the LB medium. The bacterial biosensor chosen for the preliminary screening of AHL in this study was *Chromobacterium violaceum* CV026, which responds by induction of purple violacein pigmentation [19]. The positive and negative controls for the screening were *Erwinia carotovora* GS101 and *E. carotovora* PNP22, respectively. *C. violaceum* CV026, *E. carotovora* GS101 and *E. carotovora* PNP22 were routinely cultured in LB medium. Isolate T33 was screened for AHL production by cross streaking the bacteria cultures with *C. violaceum* CV026 on LB agar plates and incubating overnight (24 h) at 28 °C. After incubation, the observed purple violacein pigmentation indicated the production of AHL by strain T33.

### 2.3. Bacteria Strain Identification

Amplification of bacterial 16S rDNA genes by polymerase chain reaction (PCR) was carried out to identify the bacteria strain according to a published method [20]. Genomic DNA was extracted using MasterPureTM DNA Purification Kit (Epicentre Inc., Madison, WI, USA). The PCR amplification and purification processes were conducted as described previously [20]. PCR product sequence alignment was done using GenBank BLASTN program, followed by phylogenetic analysis using the Molecular Evolutionary Genetic analysis version 6.0 [21,22].

### 2.4. AHLs Extraction

Strain T33 was cultured in LB broth buffered with 50 mM of 3-(*N*-morpholino)propanesulfonic acid (MOPS) (pH 5.5) in an incubator shaker (200 rpm, 28 °C, 18 h). The incubated culture was extracted twice with an equal volume of acidified (0.1% v/v glacial acetic acid) ethyl acetate as previously described [23]. The organic layer was air-dried completely and the extract was resuspended with 1 mL of acidified ethyl acetate and desiccated completely followed by addition of 200 μL of acetonitrile (HPLC grade) and vortexed to dissolve the dried extracts completely.

### 2.5. AHL Profiling by Mass Spectrometry (MS)

An Agilent RRLC 1200 system (Agilent Technologies, Agilent Inc., California, CA, USA) was utilized as the liquid chromatography (LC) delivery system with the use of an Agilent ZORBAX Rapid Resolution HT column (2.1 mm × 100 mm, 1.8 μm of particle size) for separation of AHL molecules and an Agilent 6500 Q-TOF LC/MS system was used for MS analysis. The mobile phases, injection volume, parameters for the MS analysis, ESI-positive mode, precursor ion scan mode targeting at the production ion with *m/z* 102, *m/z* value range (*m/z* 150–400) and Agilent MassHunter software for MS spectra analysis were performed essentially as reported [24].

### 2.6. Biofilm Assay

The biofilm assay was performed as described previously [25,26]. The overnight culture of strain T33 was diluted with LB medium and adjusted to OD_600_ of 0.1. Next, 50 μL of the diluted culture was added to 930 μL of LB medium supplemented with 1, 2, and 3 mg/mL of catechin, an anti-QS compound [25], in a microtitre plate. The T33 cultures were treated with and without DMSO (solvent) and served as negative and positive controls, respectively and were incubated statically for 72 h at 28 °C. The planktonic bacteria were removed by washing three times with sterile distilled water [27] and the plate was air-dried for 15 min and was stained with 0.1% (w/v) crystal violet (200 μL per well) for 30 min. Excess crystal violet was removed and the wells were washed with sterile distilled water twice followed by addition of 95% (v/v) ethanol (200 μL) and 100 μL of the resulting solution was transferred to a new, sterile microtitre plate. The absorbance of the solution was read at OD590 with microplate reader. All experiments were repeated twice.

## 3. Results and Discussion

### 3.1. Strains Isolation and Preliminary Screening of AHL

The aim of this study was to isolate the AHL-producing bacteria from a Malaysian tropical marine water sample. The sampling spot for this study was Morib Beach, a recreational attraction area. The water sample collected at the sampling spot had a temperature of 27 °C and the pH was 8.0. The marine waster sample was collected near the coastal line to determine the presence of bacteria.

**Figure 1.**
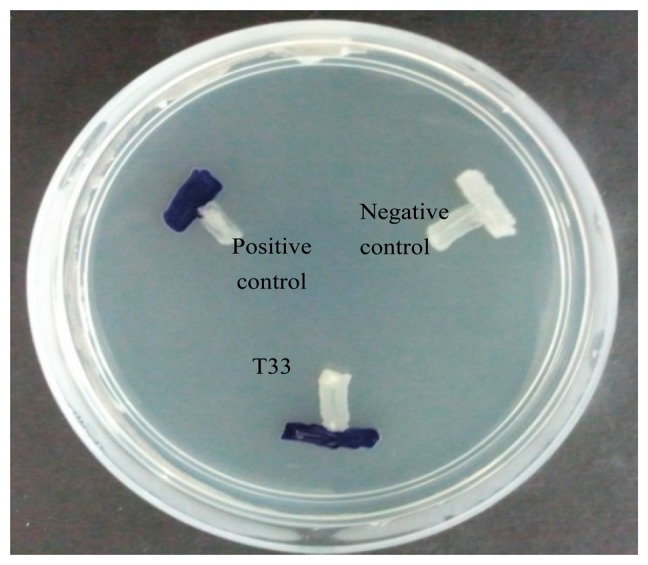
AHL screening of strain T33 with *C. violaceum* CV026. *E. carotovora* PNP22 (negative control) devoid of QS activity was included and *E. carotovora* GS101 (positive control) that can activate CV026 was included for comparison.

The availability of AHL biosensors increases the capability of researchers to discover samples of QS bacteria present [3,28]. A AHL biosensor such as *C. violaceum* CV026 is a mutant with defective LuxI AHL synthase, that practically depends on the LuxR protein in displaying specificity binding towards the cognate AHL that is able to activate the transcription of the reporter gene [19,29,30]. The biosensor *C. violaceum* CV026, responds to AHLs with C4 to C8 acyl chain length that will induce a purple violacein pigmentation [19]. It is the preferable biosensor for AHL preliminary screening due to the speed and accuracy in AHL detection, hence, we employed *C. violaceum* CV026 for the preliminary screening of AHLs produced by strain T33 (Figure 1).

The preliminary screening of strain T33 showed a positive result whereby *C. violaceum* CV026 produced a purple violacein pigmentation. This further indicates that the isolated strain T33 produces short chain AHLs. This strain was then subjected to molecular identification and AHL profiling by mass spectrometry.

### 3.2. Molecular Identification of Bacterial Strain

The identity of the isolate T33 was confirmed by analysis of its 16S rDNA gene nucleotides sequences showing that it clusters closely to the *Vibrio* genus where the strain shared 99% similarity in the BLAST search.

**Figure 2.**
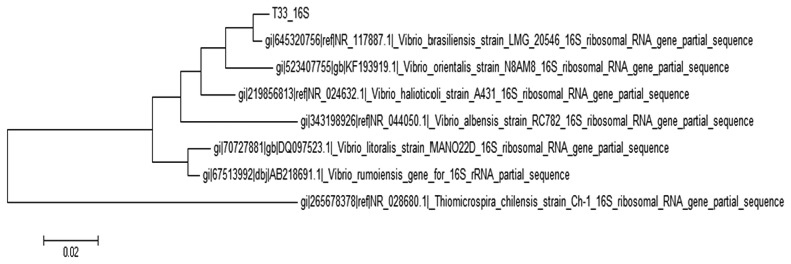
Phylogenetics analysis of strain T33. The tree is drawn to scale, and branch lengths represents number of base substitutions per site. There were a total of 1244 unambiguous nucleotides used for analysis using MEGA6.

According to the phylogenetic tree constructed (Figure 2), strain T33 was identified as *Vibrio brasiliensis*, a marine bacterium. The evolutionary history was inferred by using the Maximum Likelihood method based on the Tamura-Nei model by using Neighbor-Join and BioNJ algorithms.

### 3.3. Identification of AHL Production

The *Vibrio* genus is a causative agent of different food-borne diseases and in many countries it is a major foodborne pathogen, especially when improperly handled seafood is consumed and mortality related to *Vibrio* sp. has been reported [14]. However, the pathogenicity of *Vibrio brasiliensis* has so far not been documented. Our work has shown for the first time that this bacterium actually exhibits a QS mechanism that regulates certain physiological activities of *V. brasiliensis*.

**Figure 3.**
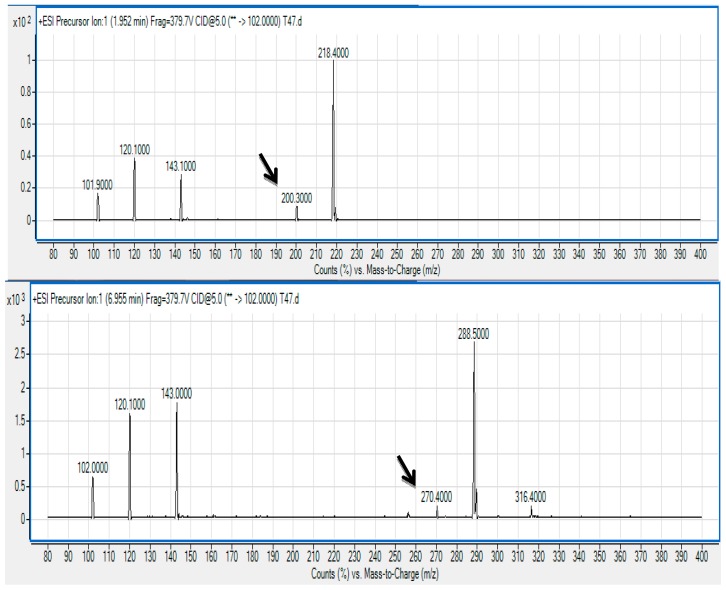
Mass spectrometry analysis of spent supernatants extract of *V. brasiliensis* strain T33. Upper Panel: mass spectrum of C6-HSL (*m/z* 200.3000; retention time 1.952 min) (marked by arrow); Lower Panel: mass spectrum of 3-oxo-C10 HSL (*m/z* 270.4000; retention time 6.955 min) (marked by arrow).

The spent culture supernatant of *V. brasiliensis* T33 strain was analyzed using the Agilent 6500 Q-TOF LC/MS system and mass spectrometry analysis. The presence of *N*-hexanoyl-l-homoserine lactone (C6-HSL) (*m/z* 200.3000; retention time 1.952 min) and *N*-(3-oxodecanoyl)-l-homoserine lactone (3-oxo-C10 HSL) (*m/z* 200.3000; retention time 6.955 min) was identified (Figure 3) and confirmed by comparing the retention times of the AHLs produced by T33 with the standard retention times as mentioned previously [24].

The precursor ion scan mode targeting at the production ion with *m/z* 102 indicates the presence of the core lactone ring moiety [31,32]. To the best of our knowledge, this is the first documentation on AHL profiling of *V. brasiliensis* where it produces these AHL molecules. This result paves the way for research towards a deeper approach into studying the mechanism on QS of *V. brasiliensis* such as characterising the *luxI* and *luxR* homologues of this isolate. We are currently conducting whole genome sequencing on *Vibrio* sp. strain T33 aiming to isolate the AHL synthase and receptor genes that will provide more insight into the QS regulatory system in this bacterium.

A battery of physiological activities such as biofilm formation, virulence factors and motility can be regulated by QS [33] and hence this work provided evidence to illustrate the significance of the research on AHL-producing bacteria present in environmental samples such as marine waters. Isolation of QS bacteria from marine water may indicate that it could be a potential reservoir for QS bacteria and more intense research should be conducted to address this issue.

### 3.4. Biofilm Formation of Vibrio sp. Strain T33

**Figure 4.**
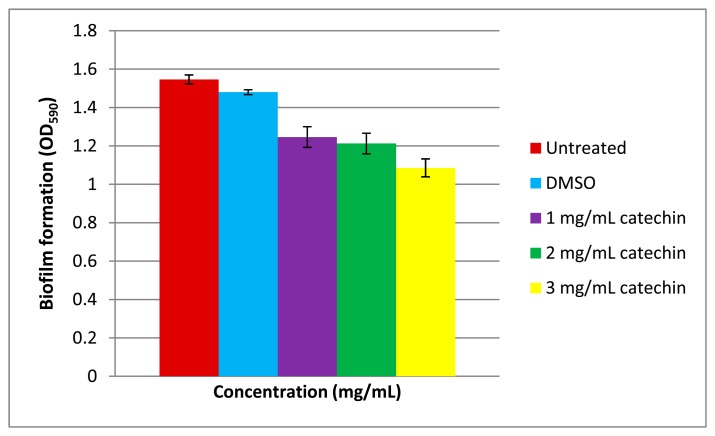
Biofilm formation in *Vibrio* sp. strain T33. Qualitative analyses of biofilm formation and inhibition by catechin. Bars represent standard errors of the mean.

Biofilm formation is often a QS-regulated phenotype [26,27] that is a multiple-stage process involving initial colonization, attachment to a surface, maturation and occasionally dispersion. The ability of bacteria to form biofilms is often linked to pathogenicity. It has been well-documented that members of *Vibrio* form biofilms [34,35]. In this work, *Vibrio* sp. strain T33 has been shown to form biofilms too (Figure 4). Under our experimental conditions, catechin reduced the biofilm formation of *Vibrio* sp. strain T33 in a dose-dependent manner. In our study, we used the well-known anti-QS compound namely catechin [36] which effectively inhibited the biofilm formation by *Vibrio* sp. strain T33. Since QS is a regulatory system for the expression of myriad virulence factors [15], this work illustrated formation of biofilm in our *Vibrio* isolate is regulated by QS. This work also suggests that marine seawater may be a potential reservoir for QS pathogens that should be given appropriate attention as it might be a possible threat to the aquaculture industry.

## 4. Conclusions

We have reported the unique AHL profile of *V. brasiliensis* strain T33 isolated from a marine water sample. Two AHLS, namely C6-HSL and 3-oxo-C10 HSL, were extracted and identified from the spent culture supernatant. To the best of our knowledge, this is the first documentation of the fact *Vibrio brasiliensis* produces these two AHLs.
